# Influenza vaccination coverage against seasonal and pandemic influenza and their determinants in France: a cross-sectional survey

**DOI:** 10.1186/1471-2458-11-30

**Published:** 2011-01-12

**Authors:** Sophie Vaux, Dieter Van Cauteren, Jean-Paul Guthmann, Yann Le Strat, Véronique Vaillant, Henriette de Valk, Daniel Lévy-Bruhl

**Affiliations:** 1 Department of infectious diseases. Institut de Veille Sanitaire (InVS) (French Institute for Public Health Surveillance), Saint-Maurice, France

## Abstract

**Background:**

Following the emergence of the influenza A(H1N1)2009 virus, the French ministry of health decided to offer free vaccination against pandemic influenza to the entire French population. Groups of people were defined and prioritised for vaccination.

**Methods:**

We took a random sample of the population of mainland France and conducted a retrospective cross-sectional telephone survey to estimate vaccination coverage against seasonal and pandemic influenza and to identify determinants of these vaccinations.

**Results:**

10,091 people were included in the survey. Overall seasonal influenza vaccination coverage (IVC) remained stable in the population from the 2008-2009 season to the 2009-2010 season reaching 20.6% and 20.8% respectively. Overall pandemic IVC in the French population is estimated to be 11.1% (CI95%: 9.8 - 12.4). The highest pandemic IVC was observed in the 0-4 years age group. For individuals with health conditions associated with higher risk of influenza, pandemic IVC was estimated to be 12.2% (CI95%: 9.8 - 15.1). The main determinants associated with pandemic influenza vaccine uptake were: living in a household with a child < 5 years OR_adj_: 2.0 (CI95%: 1.3 - 3.1) or with two children < 5 years or more, OR_adj_: 2.7 (CI95%: 1.4 - 5.1), living in a household where the head of the family is university graduate (>2 years), OR_adj_: 2.5 (CI95%: 1.5 - 4.1), or has a higher professional and managerial occupation, OR_adj_: 3.0 (CI95%: 1.5 - 5.5) and being vaccinated against seasonal influenza, OR_adj_: 7.1 (CI95%: 5.1 - 10.0). Being an individual with higher risk for influenza was not a determinant for pandemic influenza vaccine uptake. These determinants are not the same as those for seasonal influenza vaccination.

**Conclusions:**

Overall A(H1N1)2009 influenza vaccine uptake remained low, particularly among individuals with higher risk for influenza and was lower than that observed for seasonal influenza. The reasons behind people's reluctance to be vaccinated need to be investigated further.

## Background

Influenza virus infection is a major public health problem, as shown by its high morbidity and mortality. On 11 June 2009, the outbreak of the influenza A(H1N1) virus was declared a pandemic by the World Health Organization [1]. The first case of A(H1N1)2009 in France was reported on 1 May 2009 [2]. In the weeks that followed, an increase in cases was observed and the containment phase was declared over on 6 July 2009 [3]. The virus spread through mainland France during the summer, and the pandemic wave lasted from late October to late December 2009 [4].

Currently, the French recommendations for yearly seasonal influenza include vaccination of all people over 64 years, people with specific underlying diseases, and the professionals who are in contact with them, including health care workers (HCW).

These recommendations were maintained for the 2009-2010 season because co-circulation of seasonal viruses with A(H1N1)2009 virus could not be excluded. In France, seasonal influenza vaccination is provided free of charge to all individuals belonging to the risk groups. Each year, they receive a voucher for free-of-charge seasonal vaccination from the national health insurance fund.

In 2009, the ministry of health offered free pandemic influenza vaccination to the entire French population. Priority groups for vaccination were defined and ranked (Table 1) [5]. Individuals were identified by the National Health Insurance fund and a voucher for free vaccination was sent to the different groups one by one. This decision was made because the vaccine was not immediately available for the entire population. The vaccination campaign for health care workers began in late October, and the campaign for the rest of the population in mid-November. A(H1N1) pandemic vaccination was carried out in special vaccination centres specifically set-up for logistic reasons, and in order to avoid overloading general practitioners and paediatricians in case of high demand for care for A(H1N1)2009 patients.

**Table 1:** Priority groups for pandemic vaccination. Adapted from [5]

Groups	Priority
Health care workers and emergency service personnel in contact with influenza cases or at-risk individuals for influenza, pregnant women, infants aged 6-23 months at risk of influenza complications, household contacts and caregivers for infant younger than 6 months of age	1
Individuals aged 2-64 years at risk of influenza complications	2
Infants aged 6-23 months without risk factors for influenza, individuals > 64 years at risk of influenza complications	3
Individuals aged 2-18 years without risk factors for influenza	4
Individuals > 18 years without risk factors for influenza	5

Before the start of the pandemic, the French Institute for Public Health Surveillance (InVS) organized a population-level telephone survey that would estimate the population prevalence of gastrointestinal illness and self-defined influenza between May 2009 and April 2010. The telephone survey would also estimate seasonal influenza vaccination coverage and identify determinants of vaccination uptake. In the context of the pandemic, the questionnaire was adapted to obtain real-time estimates of influenza vaccination coverage for both seasonal and pandemic influenza and also to identify possible determinants of pandemic influenza vaccine uptake.

This article presents the results relating to influenza vaccination coverage (IVC) and the determinants of vaccination uptake.

## Methods

The retrospective cross-sectional telephone survey was carried out between May 2009 and April 2010 among a random sample of the French mainland population (the French overseas territories are not included in this study).

### Study population and sample

The study population included all people living in residential households in mainland France who were connected to a land telephone line and who spoke French. Households and household members were randomly selected for interview. At the first level, the sampling frame was the French mainland telephone directory stratified by region and town size. Each month a list of around 2750 numbers was selected randomly from the French telephone directory. Each number was then incremented by one, in order to generate a list including also unlisted telephone numbers. This new list replaced the first one.

At the second level, the sampling frame was the residents of the selected household. Persons were stratified by age (< 5 years, ≥ 5 years), and one child < 5 years (if any), and one person ≥ 5 years were randomly selected among the family members by taking the person who had the next birthday. If the selected child was < 18 years and ≥ 12 years old, a parent could choose to answer for the child or allow the child to answer. If the child was < 12 years old, one parent was asked to answer for him.

In order to reduce refusals, a letter introducing the survey was sent by post before the interview to the households listed in the telephone directory. Interviews were conducted from 4 pm to 9 pm from Monday to Friday and from 10 am to 2 pm on Saturdays, during two weeks each month. As many as 7 rings per call and 20 calls were made to each sampled phone number. If the selected person was not available, a telephone appointment was offered. All non-residential households, such as offices, institutions or holiday homes, were excluded from the study. Each month approximately 840 individuals were included.

All interviews were conducted by professional interviewers, using Computer Assisted Telephone Interviews (CATI). The interviewers were monitored by supervisors to assess adherence to surveying standards. The interviewers-to-supervisor ratio was 6:1. Daily quality controls were performed by supervisors.

The sample size of 800 questionnaires per month was calculated to obtain accurate estimates for incidence of gastrointestinal illness and of self-defined influenza, which were the main outcomes of the survey. Concerning influenza vaccination coverage, this sample size allowed a precision of 5% for an overall IVC of 50%, with an α error of 5% and a conservative hypothesis of a design effect of 2.

### Data collection

Verbal consent was obtained. If a child was selected, consent for participation in the study was requested from a parent (or legal guardian). If the selected resident declined to participate, an attempt was made to collect possible reasons of refusal.

The questionnaire was pilot-tested for clarity and length with 169 interviews in March-April 2009.

Questions were related to seasonal influenza vaccination uptake during the 2008-2009 season and during the 2009-2010 season according to the date of the interview (before or after September 2009); to whether or not individuals were likely to be vaccinated free of charge for seasonal vaccination, receipt of a personal voucher from the national health insurance fund; to pandemic influenza vaccination uptake and to whether or not the respondent had received a personal voucher from the national health insurance to be vaccinated for pandemic influenza at the time of the survey.

The gender and age of each respondent were collected, as well as socio-demographic characteristics of the household: household size and age of persons living in the household, education level and occupation of the head of the family. Adults were asked whether they were healthcare workers. Women were asked whether they were pregnant (question introduced in December 2009).

According to national regulations, ethical approval was not required for this observational retrospective study [6]. However, a verbal consent was obtained for the interview and all data transmitted to InVS were anonymous.

### Analysis

**Figure 1:**
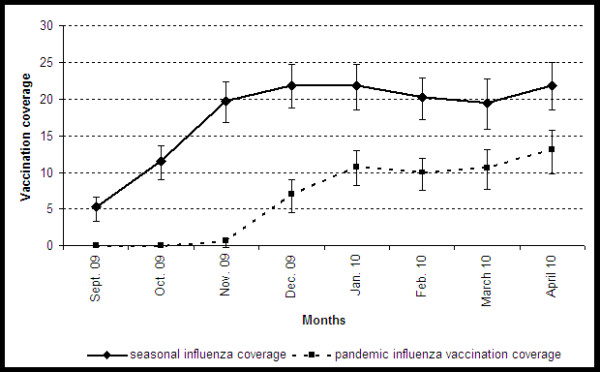
**Monthly estimates of influenza vaccination coverage (CI95%) for seasonal and pandemic influenza from September 2009 to April 2010**.

The main outcomes were seasonal and pandemic IVC. Seasonal IVC for the 2008-2009 season was estimated from interviews carried out between May and August 2009. Seasonal and pandemic IVC for the 2009-2010 season were estimated from interviews carried out from January 2010 onwards when monthly IVC reached a plateau (Figure 1). An at-risk individual of seasonal influenza was defined as a person who reported having received a personal voucher for free seasonal vaccination from the national health insurance fund. All estimates took into account the sampling design components (primary sampling unit, sampling weights) in all calculations (descriptive analyses, confidence intervals, logistic regressions). For each respondent, sampling weights were adjusted by age, gender, region and town size. For health care workers, the analysis took into account people aged 20-64 years. Possible determinants of seasonal and pandemic influenza vaccination uptake were investigated using univariate and multivariate logistic regression. Explanatory variables tested were: age, gender, number of children < 5 years and number of persons in the household, town size, being an at-risk individual, region of residence, educational level and occupation of the head of the family, access to free vaccination for seasonal influenza and the seasonal influenza vaccine uptake for pandemic vaccination. All exposures where the association showed a p value < 0.2 in the univariate analysis were included and forced in the multivariate model. A two-sided p value < 0.05 was considered to be statistically significant. Odds ratios, adjusted odds ratios and 95% confidence intervals (95% CI) are presented for the main findings. Only statistically significant variables in the univariate analysis (p < 0.2) are listed.

We tested additional interaction effects (age groups and gender, at-risk individual and gender) with a two-sided p value < 0.05 considered to be statistically significant. Collinearity between variables was tested. Data analyses were performed using Stata 9.2^® ^(StataCorp, Texas, USA).

## Results

### Participation

Of the 32,676 phone numbers selected, contact was established with 17,036 but 1,053 phone numbers were excluded because they did not correspond to a residential household. Among the 15,983 households eligible for the survey, 8,905 accepted to participate (response rate: 55.7%). Reasons given for refusals were (more than one response possible): lack of time (42%), not interested in the survey (42%), never take part in interviews (21%). A total of 10,130 people were randomly selected, of which 10,091 were included in the survey.

### Seasonal and pandemic influenza vaccination coverage

The estimated IVC for the 2008-09 season is based on the 3,399 interviews made from April to August 2009, while the estimates for seasonal and pandemic IVC for the 2009-2010 season are based on the 3,264 interviews made from January to April 2010.

Overall seasonal IVC remained stable within the population from the 2008-2009 season to the 2009-2010 season, reaching 20.6% and 20.8% respectively (Table 2). Analysis by age groups did not show any statistical differences between the two seasons. The highest seasonal IVC was observed among adults older than 64 years. Among at-risk individuals, the overall seasonal IVC was estimated at 54.9% in 2008-2009 and remained stable in the following season (54.5%). Analysis by age groups did not show any differences, but confidence intervals were large. Seasonal IVC for HCW showed an increasing trend, with 33.9% in 2009-10 compared with 24.9% (N = 133 interviews) in 2008-2009, but this difference is not statistically significant.

**Table 2:** Estimates of IVC for seasonal influenza (2008-2009 and 2009-2010 seasons) and pandemic influenza (2009-2010 season)

	**Seasonal influenza**				**A(H1N1)2009 influenza**	
		
	**2008-2009 season**		**2009-2010 season**		**2009-2010 season**	
			
	N*	**IVC** **(95% CI)**	N*	**IVC** **(95% CI)**	N*	**IVC** **(95% CI)**
			
**Age group**						
0 - 4 years	395	9.9 (6.8 - 14.2)	410	4.9 (2.9 - 8.0)	407	17.6 (14.1 - 21.8)
5 - 14 years	345	6.5 (4.0 - 10.3)	371	7.0 (4.5 - 10.6)	371	12.7 (9.4 - 17.0)
15 - 29 years	423	8.9 (6.1 - 12.8)	371	8.1 (5.4 - 12.1)	371	6.6 (4.2 - 10.2)
30 - 64 years	1,506	15.9 (14.1 - 17.9)	1,555	17.3 (15.4 - 19.5)	1,555	11.7 (10.0 - 13.6)
Older than 64 years	730	62.7 (58.9 - 66.4)	557	62.6 (58.1 - 66.9)	557	10.4 (8.0 - 13.6)
**All age groups**	**3,399**	**20.6** **(19.2 - 22.1)**	**3,264**	**20.8** **(19.3 - 22.4)**	**3,261**	**11.1** **(9.8 - 12.4)**
**Healthcare workers**	**133**	**24.9** **(17.9 - 33.5)**	**120**	**33.9** **(25.4 - 43.6)**	**120**	**29.8** **(21.7 - 39.4)**
**Gender**						
Male	1,522	21.5 (19.3 - 23.9)	1,449	19.2 (17.0 - 21.6)	1,449	9.2 (7.9 - 10.2)
Female	1,877	19.8 (18.0 - 21.7)	1,815	22.3 (20.3 - 24.5)	1,812	7.8 (6.7 - 9.1)
**At-risk individuals**						
0 - 4 years	22	31.7 (13.4 - 58.2)	32	18.6 (7.6 - 38.8)	32	25.3 (11.6 - 46.5)
5 - 14 years	14	13.1 (4.0 - 35.4)	27	31.5 (15.1 - 58.9)	27	21.5 (8.3 - 45.6)
15 - 29 years	23	21.8 (7.4 - 49.2)	20	24.3 (6.7 - 51.1)	20	0 (-)
30 - 64 years	133	40.3 (31.7 - 50.0)	150	41.1 (32.5 - 50.3)	150	17.9 (12.2 - 25.6)
Older than 64 years	730	62.7 (58.9 - 66.4)	557	62.6 (58.1 - 67.0)	557	10.4 (7.9 - 13.6)
All age groups	922	54.9 (51.2 - 58.6)	786	54.5 (50.6 - 58.4)	786	12.2 (9.8 - 15.1)
Younger than 65 years	192	33.6 (26.2 - 42.0)	229	35.5 (28.0 - 43.8)	229	16.3 (11.4 - 22.8)
**Pregnant women**	-	-	71	5.4 (1.8 - 15.4)	71	12.8 (5.7 - 26.1)

*N: number of respondents

Overall pandemic IVC in the French population was 11.1% (CI95%: 9.8 - 12.4). The pandemic IVC in the 0-4 years age group was significantly higher than the seasonal IVC in the same age group (17.6% vs. 4.9%). The estimate of pandemic IVC for children younger than 3 years was 19.2% (CI95%: 14.3 - 25.2). Pandemic IVC in those older than 64 years was 10.4% (CI95%: 8.0 - 13.6), significantly lower than the seasonal IVC in this age group (63%). For at risk individuals, overall pandemic IVC was estimated at 12.2% (CI95%: 9.8 - 15.1) and IVC for those younger than 65 years at 16.3% (11.4 - 22.8). Both were significantly lower than the IVC estimated for seasonal influenza.

For HCW, pandemic and seasonal IVC were comparable for the 2009-2010 season (29.8% and 33.9% respectively). For pregnant women, who were not targeted for seasonal influenza vaccination, pandemic IVC was low, at 12.8% (CI95%: 5.7 - 26.1).

The proportion of people who reported having received a personal voucher for free pandemic influenza vaccination increased with time. These proportions were: 13.3% (CI95%: 10.8 - 16.3) in November 2009, 33.2% (CI95%: 29.6 - 37.0) in December 2009, 67.4% (CI95%: 63.8 - 70.8) in January 2010, and 86.3% (CI95%: 83.6 - 88.6) in February, and remained relatively stable during following months. Analysis by age group showed that in January 2010, when the pandemic wave was over in mainland France, receipt of these vouchers was estimated at 83.2% (CI95%: 74.8 - 89.2) for the 0-4 years age group, 69.2% (CI95%:59.0 - 77.8) for the 15-29 years age group and 47.3% (CI95%: 38.6 - 56.2) for adults older than 64 years.

### Determinants of influenza vaccination coverage

Analyses of determinants were based on interviews made from January to April 2010. For seasonal IVC, multivariate analyses showed that being an at-risk individual for influenza, being a HCW, living in a household where the head of the family was retired or living in an urban area were predictive of a greater vaccination uptake (Table 3). Being younger than 30 years old and living in a household where the head of family was university graduated were predictive of a lower seasonal vaccination uptake. The number of persons in the household, the number of children < 5 years and the gender were not significantly associated with seasonal vaccination uptake.

**Table 3:** Potential determinants for IVC for seasonal and pandemic influenza, 2009-2010 season

	**Seasonal influenza vaccination**			**Pandemic influenza vaccination**		
	**Odd ratio (95% CI)**			**Odd ratio (95% CI)**		
		
	**Unadjusted univariate**	**Adjusted multi-variate**	***p*-value**	**Unadjusted univariate**	**Adjusted multi-variate**	***p*-value**
		
**Age group** (Ref. Older than 64 years)						
0 - 4 years	0.03 (0.02-0.05)	0.3 (0.1-0.6)	< 0.001	1.8 (1.2-2.7)	2.1 (1.0-4.5)	0.04
5 - 14 years	0.04 (0.03-0.07)	0.3 (0.2-0.6)	0.001	1.2 (0.8-2.0)	2.2 (1.0-4.8)	ns (0.05)
15 - 29 years	0.05 (0.03-0.09)	0.4 (0.2-0.8)	0.005	0.6 (0.3-1.1)	1.3 (0.5-3.0)	Ns
30 - 64 years	0.13 (0.1-0.2)	0.8 (0.5-1.2)	0.25	1.1 (0.8-1.6)	2.0 (1.0-3.8)	0.04
**Number of children (< 5 years) in the household **(ref. None)						
One child	0.2 (0.2-0.4)	0.8 (0.5 - 1.3)	ns	2.0 (1.4-2.9)	2.0 (1.3-3.1)	0.003
Two children or more	0.3 (0.1-0.6)	0.8 (0.3 - 1.7)	ns	2.5 (1.4-4.4)	2.7 (1.4-5.1)	0.004
**Number of persons in the household** (ref: 1 person)						
2 persons	0.8 (0.7-1.0)	0.9 (0.7 - 1.2)	ns	1.5 (1.0-2,1)	1.5 (1.0-2.2)	Ns
3 persons	0.23 (0.2-0.3)	0.8 (0.5 - 1.1)	ns	2.0 (1.3-3.1)	3.5 (1.5-4.1)	< 0.001
4 persons or more	0.2 (0.2-0.3)	0.8 (0.5 - 1.2)	ns	2.2 (1.5-3.2)	3.5 (1.4-4.3)	0.001
**Gender** **(Ref. female)**	0.8 (0.7-1.0)	1.1 (0.9 - 1.4)	ns	1.2 (0.9-1.5)	1.1 (0.8-1.4)	Ns
**Education level of the head of the family** **(ref. primary level of education)**						
Less than high school	0.3 (0.2-0.4)	0.7 (0.5 - 1.0)	ns	1.0 (0.7-1.5)	1.3 (0.8-2.0)	Ns
High school graduate	0.3 (0.2 - 0.4)	0.7 (0.5 - 1.1)	ns	0.8 (0.4-1.3)	0.8 (0.4-1.4)	Ns
University graduate (2 years)	0.1 (0.1-0.2)	0.4 (0.2 - 0.7)	< 0.001	1.3 (0.8-2.2)	1.5 (0.9-2.7)	Ns
University graduate (>2 years)	0.3 (0.2-0.4)	0.9 (0.6 - 1.3)	ns	3.0 (2.0-4.4)	2.5 (1.5-4.1)	< 0.001
**At-risk individual** (Ref. not to be an at-risk individual						
All age groups included	9.9 (7.7-12.2)	4.0 (2.6-6.0)	< 0.001	1.5 (1.0-2.3)	1.2 (0.7-2.0)	Ns
At-risk individual younger than 65 years old	4.5 (3.1-6.6)	4.0 (2.6-6.1)	< 0.001	1.6 (1.0-2.5)	1.2 (0.7-2.0)	Ns
Healthcare worker*	4.2 (2.6-6.5)	4.9 (2.9-8.1)	< 0.001	4.4 (2.7-7.1)	3.0 (1.8-5.0)	0.001
**Occupation of the head of the family** (Ref. manual worker)						
Farmer	0.6 (0.2-1.6)	0.7 (0.3-1.7)	ns	2.0 (0.8-5.4)	3.0 (1.1-8.0)	0.03
Self employed	1.4 (0.8-2.5)	1.5 (0.8-2.6)	ns	2.3 (1.2-4.5)	1.9 (0.9-3.8)	Ns
Higher professional and managerial occupation	0.9 (0.5-1.4)	0.9 (0.5-1.5)	ns	3.9 (2.4-6.2)	3.0 (1.5 - 5.5)	0.001
Intermediate occupation	1.0 (0.6-1.6)	1.1 (0.6-1.9)	ns	2.2 (1.3-3.8)	2.0 (1.0-3.8)	0.04
Clerical	0.7 (0.5-1.2)	0.8 (0.5-1.3)	ns	1.3 (0.8-2.1)	1.5 (0.8-2.6)	Ns
Retired	6.9 (4.8-9.9)	1.7 (1.1-2.6)	0.02	2.2 (1.3-3.8)	2.0 (1.0-4.0)	0.04
Student	0.4 (0.1-1.9)	0.4 (0.1-2.2)	ns	1.0 (0.2-4.7)	2.3 (0.4-13.0)	Ns
Unemployed	3.1 (1.7-5.7)	1.9 (0.9-3.7)	ns	0.8 (0.3-2.0)	0.7 (0.2-1.9)	Ns
**Town size** (Ref. rural)						
< 20,000 inhabitants	1.4 (1.0-1.8)	1.4 (1.0-1.9)	0.03	1.0 (0.7-1.5)	-	
[20,000; 100 000[ inhabitants	1.4 (1.0-1.9)	1.5 (1.1-2.2)	0.02	1.2 (0.7-1.8)	-	
≥ 100 000 inhabitants	1.3 (1.0-1.6)	1.6 (1.2-2.2)	< 0.001	1.3 (0.9-1.9)	-	
Paris	0.9 (0.7-1.3)	1.2 (0.8-1.7)	ns	1.4 (0.9-2.2)	-	
**Free seasonal influenza vaccination offered (for non at-risk individuals)**	3.9 (2.9-5.3)	4.5 (3.2-6.3)	< 0.001	-	-	
**Vaccinated against seasonal influenza**	-	-	-	3.5 (2.7-4.6)	7.1 (5.1-10.0)	< 0.001

* for people aged 20-64 years

ns: non significant

For pandemic IVC, the following factors were predictive of greater vaccination uptake after multivariate analyses: belonging to the 0-4 years age-group, to the 30-64 years age-group, living in a household with one or more children aged < 5 years, with 2 or more persons, where the head of the family is university graduated (>2 years), or where the head of the household was a farmer, has a higher professional and managerial occupation, has an intermediate occupation or was retired (compared with being a manual worker).

When the analysis was restricted to households without children younger than 5 years old, the size of the household remained a determinant to increase vaccination uptake: 3 persons in the household, OR_adj_: 2.2 (CI95%: 1.3 - 3.9, p = 0.006); 4 persons or more in the household, OR_adj_: 2.6 (CI95%: 1.4 - 4.7, p = 0.003).

People vaccinated against seasonal influenza were more likely to be vaccinated against pandemic influenza (OR_adj_: 7.1 (5.1 - 10.0), p < 0.001).

No significant association was found between the pandemic vaccination coverage and being a subject at risk of influenza complications, the town size or gender.

## Discussion

Overall A(H1N1)2009 pandemic influenza vaccine uptake was low at 11.1% (CI95%: 9.8 - 12.4). The main result of the study is the observed low influenza vaccine uptake of 16.3%(95% CI 11.4-22.8) among individuals less than 65 years old who are at-risk for complications due to influenza infection. This IVC is considerably lower than the seasonal IVC in this same group (35.5%). Contrary to seasonal IVC, being at-risk for influenza was not associated with higher pandemic vaccine uptake.

In France, 93% of A(H1N1)2009 influenza severe cases were younger than 65 years, and 53% had an underlying disease putting them at high risk for seasonal influenza [7].

Early in the pandemic, pregnancy was considered to be a risk factor for A(H1N1)2009 influenza complications [8], and therefore pregnant women were included in the first priority group to be invited for pandemic vaccination. Although public health experts strongly recommended vaccination for pregnant women, pandemic IVC remained low in this population (12.8%). This was despite the results of a survey conducted in November 2009, that found that 37.9% of pregnant women and 34.8% of at-risk individuals for influenza complications intended to be vaccinated against pandemic influenza, i.e. a much higher figure than the IVC actually estimated through our survey [9].

The highest pandemic IVC was observed among children younger than 5 years old, who were one of the first groups to be invited for pandemic vaccination. An increased pandemic influenza uptake was associated with the number of children younger than 5 years old in the household and with the number of persons in the household. These determinants are not found for seasonal influenza. This suggests that compliance with the pandemic vaccination campaign was higher in families with children.

Low pandemic vaccine uptake (10.4%) was observed in persons older than 64 years, which is much lower than the seasonal vaccine uptake (62.6%). Adults older than 64 years old without risk factors for influenza were the last group to receive their voucher in January 2010, when the epidemic wave was over in mainland France. As a consequence, less than half of the people in this age group reported having received the pandemic vaccination voucher in January. Fortunately, elderly people were relatively unaffected by the pandemic, presumably because of cross-protective antibodies [10]. A personal voucher for free seasonal vaccination was sent to all persons older than 64 years, making it impossible to distinguish people with underlying diseases for this age group in our survey.

WHO recommended that "All countries should immunize their health-care workers as a first priority to protect the essential health infrastructure" [11]. This recommendation was followed in France and HCW were the first group to be invited for pandemic vaccination. The vaccination campaign for this population began in health care settings (such as hospitals, clinics) before the opening of mass vaccination centres. Pandemic IVC for health care workers was estimated at around 30%. This result was dramatically lower than the expected rate (62%) estimated by a study on acceptability for A(H1N1) vaccination conducted among HCW between June and September 2009 [9]. However, analyses of determinants for IVC showed that being an health care worker is predictive of a better influenza vaccination uptake both for seasonal and for pandemic influenza.

Our study allows to compare at a national level for the same influenza season, vaccination coverage of seasonal and pandemic influenza and determinants of these vaccinations. However, this study has several limitations. First, it has the usual limitations of retrospective surveys based on self-report, such as recall bias. Second, a better participation of persons favourable to vaccination can not be avoided. Third, our study excluded households without telephone and those with only mobile-telephones but, this was in part corrected by adjusting sampling weights by age, gender, region and town size. Because of small numbers in some groups, confidence intervals were sometimes wide, explaining that results should be interpreted with caution. We believe, however, that the impact of these possible biases or limitations is likely to be limited and that the data produced by this survey contribute significantly to the knowledge of IVC. The validity of our results was supported by comparison of estimates produced by other French data sources. Seasonal IVC estimated by this survey are close to those of previous seasons produced by the French general health insurance scheme for at-risk individuals [12] and for the overall population [13]. Pandemic IVC estimates are slightly higher than those produced by official statistics [14] (5.3 millions individuals with at least one vaccination, around 8.3% of the population) and those estimated from data produced by the French general health insurance scheme (5.2 millions, around 8.0% of the population) [15]. The small discrepancies observed may be explained by the participation and reporting biases known to occur in surveys based on self-reporting, the possibility that some vaccinations could not be recorded in the general health insurance vaccination database, or a combination of both mechanisms.

In most countries where the figures are known, pandemic IVC remained low. Data collected by the French National Assembly investigating committee into the influenza A(H1N1) vaccination campaign in France noted uptakes of 10% or less in Germany, England, Belgium, Spain and Italy [16]. Higher IVC than those observed in France were reported in the United States, Sweden, Canada, the Netherlands and Japan [17,16]. In England, uptake was reported to be 37.1% in risk groups (including pregnant women) [18].

Analysis of determinants of IVC coverage for pandemic influenza in comparison with those for seasonal influenza shows differences and allows drawing some hypotheses explaining the low vaccination uptakes observed. Doubts about the severity of the A(H1N1)2009 epidemic, about the safety and effectiveness of adjuvanted pandemic vaccines approved through a fast-track procedure, and the lack of post-marketing surveillance data may explain why people were reluctant to be vaccinated. In France, as in other countries, these subjects were highly discussed in the media in the autumn of 2009. In November 2009, only one third of the French general population considered A(H1N1)2009 influenza illness to be a "severe" or "very severe disease", and respondents with a higher perception of severity of illness and a higher level of concern were significantly more likely to accept vaccination [9,19]. Results of our study suggest that a higher level of concern about pandemic influenza was observed among children and in households with the presence of children. The fact that higher pandemic IVC was observed in households where the head of the family was a farmer may be related to the origin of this new virus first reported as of swine origin.

The mass vaccination centres set up for the pandemic immunization campaign may also be a key factor explaining the low vaccination coverage. Contrary to the pandemic vaccination campaign, the administration of seasonal influenza vaccines is done by general practitioners (GPs). The role of primary care physicians as a key factor for reaching high vaccination coverage has been clearly shown [20,9]. Finally, the choice of a vaccination campaign communication targeting the whole French population, with few specific messages targeting at-risk individuals may also explain the low vaccination coverage found in our study. Uptake of seasonal influenza vaccine has been shown to be a strong predictor of vaccination intention against pandemic influenza in French and US populations [21,19]. A positive relation between seasonal and pandemic vaccine uptake was shown in our survey: individuals vaccinated against seasonal influenza were more likely to be vaccinated against pandemic influenza. Offer of a free vaccination has been shown to be a positive determinant for increasing seasonal influenza uptake, both in the general population and among health care workers [22]. However cost cannot explain the low pandemic IVC observed in France because the pandemic influenza vaccination was offered free of charge for everyone.

The reasons behind the higher pandemic IVC when the head of the family had a high education level, a managerial or intermediate occupation could reflect that these populations are more sensitive to public health recommendations or perhaps less sensitive to rumors about the lack of efficacy or safety of the vaccines. Results of our study allow to draw some hypotheses but reasons behind reluctance for pandemic vaccination needs to be further investigated in order to be better prepared for future health threats that may require mass immunisation campaigns.

## Conclusions

This nationwide study assessed influenza vaccination uptake for seasonal and pandemic influenza in the French population. Overall A(H1N1)2009 influenza vaccine uptake was low, particularly among at-risk individuals for influenza. The analysis of the determinants of influenza vaccination shows differences between both vaccination campaigns.

## Competing interests

The authors declare that they have no competing interests.

## Authors' contributions

SV, VV, HV, YLS, DLB conceived the study. SV analyzed the results in consultation with DV, VV, HV, YLS, DLB. SV wrote the draft version and revisions of the manuscript according to the contribution of DV, YLS, VV, HV, DLB. All authors read and approved the final version of the manuscript.

## Pre-publication history

The pre-publication history for this paper can be accessed here:

http://www.biomedcentral.com/1471-2458/11/30/prepub
